# Waist Circumference Modifies the Association Between a Deep Learning-Derived Retinal Biomarker and Coronary Artery Calcium Score in Asymptomatic Adults

**DOI:** 10.3390/jcm15124779

**Published:** 2026-06-19

**Authors:** Sung-Hoon Jung, Sung-Goo Kang, Sang-Wook Song, Se-Hong Kim, Dongjin Nam, Junseung Rho

**Affiliations:** 1 Department of Internal Medicine, Eunpyeong St. Mary’s Hospital, College of Medicine, The Catholic University of Korea, Seoul 03312, Republic of Korea; shjung74@catholic.ac.kr; 2 Department of Family Medicine, St. Vincent’s Hospital, College of Medicine, The Catholic University of Korea, Seoul 16247, Republic of Korea; fmssw@catholic.ac.kr (S.-W.S.); iron1600@catholic.ac.kr (S.-H.K.); mediart@catholic.ac.kr (J.R.); 3 Mediwhale Inc., Seoul 06049, Republic of Korea; jayden.nam@mediwhale.com

**Keywords:** cardiovascular risk assessment, coronary artery calcium score, deep learning, effect modification, fundus photography, subclinical atherosclerosis, visceral adiposity, waist circumference

## Abstract

**Background**: The deep learning-derived retinal cardiovascular risk index (Reti-CVD) is a deep learning-derived retinal biomarker calculated from non-mydriatic fundus photographs for cardiovascular risk assessment. This study examined whether obesity phenotype, particularly central adiposity, modifies the association between Reti-CVD and coronary artery calcium score (CACS) in asymptomatic adults undergoing routine health screening. **Methods**: We retrospectively analyzed 237 Korean adults who underwent fundus photography for Reti-CVD assessment and cardiac computed tomography for CACS measurement. Abdominal obesity was defined as waist circumference (WC) ≥ 90 cm in men and ≥85 cm in women, and general obesity as body mass index (BMI) ≥ 25 kg/m^2^. Multivariable linear regression models with sequential adjustment were used to evaluate the association between Reti-CVD and CACS. Effect modification was assessed using interaction terms for Reti-CVD×WC and Reti-CVD×BMI. Discriminatory performance for coronary calcification, defined as CACS > 0, was evaluated using the area under the receiver operating characteristic curve (AUC). **Results**: Abdominal obesity was present in 78 participants (32.9%), and general obesity in 102 (43.0%). Participants with CACS > 0 had significantly higher Reti-CVD scores than those with CACS = 0 (0.15 ± 0.09 vs. 0.09 ± 0.05; *p* < 0.001). Reti-CVD remained positively associated with CACS after adjustment for metabolic and lifestyle factors. In fully adjusted models, WC significantly moderated this association (interaction *p* = 0.0288), whereas BMI did not (interaction *p* = 0.5381). Overall discrimination for CACS > 0 was moderate (AUC = 0.735) and numerically higher in participants with abdominal obesity than in those with normal WC (0.787 vs. 0.695). **Conclusions**: Reti-CVD is independently associated with coronary calcification, and WC-based central adiposity modifies this relationship. Incorporating obesity phenotype may improve personalized interpretation of retinal biomarker-based cardiovascular risk assessment.

## 1. Introduction

Cardiovascular disease (CVD) remains a leading cause of mortality worldwide, and its burden continues to rise with population aging and shifts toward Westernized dietary patterns [1]. Early detection and risk stratification are essential to improve outcomes for people and to reduce societal costs. In clinical practice, the coronary artery calcium score (CACS) is widely used as a robust imaging biomarker of subclinical coronary atherosclerosis and future cardiovascular risk [2]. However, routine or repeated CACS assessment in primary care or large-scale screening is constrained by radiation exposure and cost [3].

Obesity is another major and growing contributor to the global cardiovascular burden. Obesity is a well-recognized risk factor for CVD. Abdominal obesity, typically assessed by waist circumference (WC), is more strongly associated with metabolic syndrome and atherosclerosis than general adiposity captured by body mass index (BMI) [4]. Visceral adiposity promotes chronic low-grade inflammation and dysregulated adipokine signaling, contributing to endothelial dysfunction [5]. Moreover, heterogeneous metabolic phenotypes exist, including metabolically healthy obesity and metabolically obese normal weight, underscoring that BMI alone may not fully capture cardiometabolic risk [6]. Therefore, WC may provide more informative risk stratification than BMI in many settings [7].

Advances in artificial intelligence (AI) have brought renewed attention to retinal imaging as a non-invasive window into systemic cardiovascular health. Retinal microvasculature can be directly visualized, and structural changes in retinal vessels may reflect early microvascular and macrovascular disease [8]. Reti-CVD, a deep learning-derived retinal biomarker computed from fundus photographs, has shown predictive performance comparable with or superior to established cardiovascular risk scores in large datasets [9,10]. Reti-CVD was originally developed using retinal photographs paired with coronary artery calcium as the ground truth, and in subsequent validation studies, it stratified future CVD risk in both Asian and European populations. In the UK Biobank, the Reti-CVD high-risk group showed an approximately 13% 10-year CVD risk and remained associated with incident CVD after adjustment for the QRISK3 score, providing incremental prognostic value when added to QRISK3 [10]. In a separate cross-sectional analysis, Reti-CVD identified individuals classified as intermediate-to-high risk by established tools (Pooled Cohort Equation, QRISK3, and the modified Framingham Risk Score) with sensitivity and specificity above 80% [9]. Despite these developments, it remains unclear whether the performance of Reti-CVD differs across metabolic characteristics, particularly obesity phenotypes.

Obesity has also been linked to retinal vascular features such as altered vessel caliber and increased tortuosity, which share mechanistic pathways with coronary artery disease [11]. However, it is not well established whether the association between Reti-CVD and CACS is stronger among people with abdominal obesity than among those with normal WC. If central adiposity amplifies the predictive value of Reti-CVD for coronary calcification, retinal biomarkers could help prioritize individuals for targeted evaluation, including cardiac CT-based CACS assessment, in screening and primary care settings [12].

The purpose of this study was to evaluate whether obesity measures (BMI and WC) moderate the association between Reti-CVD and CACS in asymptomatic Korean adults undergoing routine health screening. We compared the influence of general adiposity (BMI) and central adiposity (WC) on the predictive value of Reti-CVD using multivariable models with sequential adjustment for metabolic and lifestyle factors. By integrating obesity phenotype into retinal biomarker interpretation, we aimed to provide evidence to support more personalized cardiovascular risk stratification and prevention strategies. Clarifying this question is clinically relevant because, if central adiposity amplifies the relationship between a retinal biomarker and coronary calcification, a simple and inexpensive anthropometric measure (WC) could be combined with non-invasive fundus photography to flag individuals in whom Reti-CVD is more informative for subclinical atherosclerosis. In screening and primary care settings, where repeated CACS testing is limited by radiation exposure and cost, such a phenotype-informed approach may help prioritize who could benefit most from further cardiac evaluation, without altering established clinical thresholds.

## 2. Materials and Methods

### 2.1. Study Population

This study is a retrospective cross-sectional study of adults aged 19 years and older who underwent a comprehensive health screening at a university hospital’s health promotion center in Gyeonggi-do, Republic of Korea, between January 2024 and November 2025. Data were collected from anonymized electronic medical records.

The study included asymptomatic adults who simultaneously underwent artificial intelligence (AI)-based retinal cardiovascular risk index (Reti-CVD) assessment and coronary artery calcium scoring (CACS) on the same health-screening visit. Eligible participants were adults aged 19 years or older without cardiovascular symptoms who had complete data for both Reti-CVD and CACS, as well as for the covariates used in the analysis. To ensure objectivity and control for confounding variables, the following exclusion criteria were applied: (1) a history of moderate-to-severe cerebrovascular or cardiovascular diseases, such as myocardial infarction, angina, stroke, or prior coronary revascularization (percutaneous coronary intervention or coronary artery bypass grafting); (2) current or past medical history of malignant tumors; and (3) hyperthyroidism or hypothyroidism requiring pharmacological treatment with moderate-to-severe symptoms. Finally, data from a total of 237 participants with complete datasets were included in the analysis. This study was approved by the Institutional Review Board (IRB) of the participating medical institution. Patient consent was waived because this retrospective study analyzed de-identified data obtained during routine health screening examinations, posing minimal risk to participants. The waiver of informed consent was also approved by the Institutional Review Board of St. Vincent’s Hospital. (VC25RISI0346, Date of approval: 11 December 2025).

### 2.2. Physical Examination and Laboratory Measurements

Anthropometric measurements were performed by trained nurses following standardized protocols. Height and body weight were measured using an automated anthropometer, and body mass index (BMI) was calculated as kg/m^2^. Waist circumference (WC, cm) was measured at the midpoint between the lower rib margin and the iliac crest after gentle expiration. Abdominal obesity was defined as WC ≥ 90 cm for men and ≥85 cm for women, according to the Korean Society for the Study of Obesity (KSSO) guidelines [13]. Blood pressure (BP), including systolic (SBP) and diastolic (DBP), was measured using a mercury sphygmomanometer after at least 5 min of rest.

Venous blood samples were collected after at least 8 h of fasting. Biochemical markers included white blood cell count (WBC), liver function tests (AST, ALT), kidney function tests (Creatinine), and metabolic markers such as glycated hemoglobin (HbA1c) and low-density lipoprotein cholesterol (LDL-C).

### 2.3. Lifestyle and Medical History Questionnaire

Participants’ medical history and lifestyle habits were collected through self-administered questionnaires and medical interviews. Hypertension, diabetes mellitus, and dyslipidemia were defined based on current medication use or a physician’s diagnosis. Lifestyle variables included current smoking status, alcohol consumption, and regular aerobic exercise (defined as at least three times per week). Dyslipidemia treatment was recorded only as a categorical medication-use variable; antihypertensive and glucose-lowering medication use was likewise recorded for hypertension and diabetes, respectively, and these treatment variables were included as covariates in the fully adjusted model (Model 4). Detailed information on statin type and intensity, other lipid-lowering agents, and antiplatelet therapy was not consistently available in the routine health-screening records and was therefore not analyzed; this limitation is addressed in the Discussion section.

### 2.4. AI-Based Reti-CVD Assessment

Non-mydriatic fundus photographs of both eyes were captured using a digital fundus camera without the administration of mydriatic agents. The images were analyzed using the deep learning-based software DrNoon for CVD (Mediwhale Inc., Seoul, Republic of Korea). This system analyzes complex morphological changes in retinal vessels—including diameter, tortuosity, and arteriovenous nicking—to calculate the Reti-CVD score, a continuous value ranging from 0 to 1 that represents the risk of future cardiovascular events [9,10]. The development and validation of this algorithm have been described in detail in previous publications [9,10,12]. In brief, the underlying convolutional neural network was trained on retinal fundus photographs paired with coronary artery calcium measured by cardiac computed tomography as the ground-truth label, so that the model learned to estimate the probability of coronary artery calcification from the retinal image. The output probability score (0 to 1) was then optimized and validated as a cardiovascular risk index (Reti-CVD), with higher values indicating higher predicted risk. In the present study, the most up-to-date commercial version of the algorithm was used with default settings, and the score was generated automatically without manual adjustment. Because the algorithm is a proprietary, commercially deployed system, the exact network architecture, training weights, and internal pre-processing steps are not publicly disclosed; we therefore relied on the manufacturer’s validated pipeline and cite the original development studies for full methodological detail [9,10,12].

### 2.5. Coronary Artery Calcium Scoring

To quantify the extent of coronary artery calcification, multi-detector computed tomography (MDCT) was performed. Images were acquired using ECG-gating to synchronize with the cardiac cycle, and the calcium score was calculated using the Agatston method [2] and is expressed in Agatston units (AU). The total CACS was derived by summing the scores of the left main, left anterior descending, left circumflex, and right coronary arteries. In the study population, CACS ranged from 0 to 2384 AU. Coronary calcification was defined as a CACS greater than 0 AU. For statistical analysis, a logarithmic transformation (log[CACS + 1]) was applied to account for the skewed distribution of the data.

### 2.6. Statistical Analyses

Continuous variables are presented as mean ± standard deviation or median (interquartile range), as appropriate, and categorical variables as number (%). Between-group comparisons according to coronary artery calcium score (CACS) status (CACS = 0 vs. CACS > 0) were performed using Student’s *t*-test or the Wilcoxon rank-sum test for continuous variables and the chi-square test or Fisher’s exact test for categorical variables, as appropriate. Because CACS was right-skewed, CACS was log-transformed as log(CACS + 1) for regression analyses. To evaluate the association between Reti-CVD and coronary calcification, we fitted multivariable linear regression models with sequential adjustment using log(CACS + 1) as the dependent variable. The covariates were entered in prespecified blocks as follows: Model 1, unadjusted; Model 2, adjusted for age and sex; Model 3, additionally adjusted for hemoglobin A1c, systolic blood pressure, and white blood cell count; and Model 4, additionally adjusted for aspartate aminotransferase, alanine aminotransferase, serum creatinine, lifestyle factors (current smoking, current alcohol intake, and regular exercise), and history of hypertension, diabetes mellitus, and dyslipidemia treatment. To test whether obesity phenotype modified the Reti-CVD–CACS relationship, interaction terms (Reti-CVD×waist circumference [WC] and Reti-CVD × body mass index [BMI]) were included in Models 1–4. For phenotype-stratified analyses, abdominal obesity was defined by WC (men ≥ 90 cm; women ≥ 85 cm) and general obesity by BMI (≥25 kg/m^2^). Discrimination of Reti-CVD for the presence of coronary calcification (CACS > 0) was assessed using receiver operating characteristic (ROC) curves and the area under the ROC curve (AUC), overall and by WC phenotype. A two-sided *p*-value < 0.05 was considered statistically significant. All analyses were performed using Stata (version 14.0; StataCorp, College Station, TX, USA) and R (version 3.4.4; R Foundation for Statistical Computing, Vienna, Austria). Several additional analyses were performed to address robustness and incremental value. Because CACS is characteristically zero-inflated and right-skewed, the log(CACS + 1) transformation was used to stabilize variance and limit the influence of extreme values while retaining all participants, including those with a score of zero; the robustness of the findings to this modelling choice was further examined using alternative specifications, namely, logistic regression for the presence of coronary calcification (CACS > 0) and analyses based on clinically meaningful CACS thresholds. To address the possibility of model instability given the sample size, multicollinearity among covariates was evaluated using variance inflation factors (VIFs), and the number of events per variable was considered when interpreting the fully adjusted model. In addition to interaction P-values and adjusted R^2^, regression coefficients with 95% confidence intervals (CIs) were examined and are reported in the text to quantify the magnitude of the moderation effect. Finally, to evaluate whether Reti-CVD provided information beyond conventional risk factors, the discrimination of a baseline model comprising demographic and cardiovascular risk factors (age, sex, systolic blood pressure, hemoglobin A1c, smoking, hypertension, diabetes, dyslipidemia, and BMI) was compared with that of the same model with Reti-CVD added, using the C-statistic (AUC) and the DeLong method.

## 3. Results

### 3.1. Characteristics of the Study Participants

The demographic and clinical characteristics of the 237 participants are summarized in Table 1. The mean age was 50.8 years, and 115 participants were male (48.5%). Abdominal obesity, defined by waist circumference (WC), was present in 78 participants (32.9%), and general obesity, defined by body mass index (BMI ≥ 25 kg/m^2^), was present in 102 participants (43.0%). The mean Reti-CVD score was 0.12, and the mean coronary artery calcium score (CACS) was 45.2. Participants with CACS > 0 (*n* = 118) were older and more frequently male than those with CACS = 0 (*n* = 119) (*p* < 0.001 for both), and had higher WC (*p* = 0.001) and systolic blood pressure (*p* < 0.001). The CACS > 0 group also showed higher white blood cell count (*p* = 0.042), hemoglobin A1c (*p* < 0.001), aspartate aminotransferase (*p* = 0.038), alanine aminotransferase (*p* = 0.041), and serum creatinine (*p* = 0.012). BMI and LDL-cholesterol did not differ significantly between groups (*p* = 0.092 and *p* = 0.748, respectively). The Reti-CVD score was higher in participants with CACS > 0 than in those with CACS = 0 (0.15 ± 0.09 vs. 0.09 ± 0.05; *p* < 0.001).

### 3.2. Association Between Reti-CVD and CACS and the Moderating Effect of Obesity Indices

In simple correlation analysis, Reti-CVD was positively correlated with log-transformed CACS (log[CACS + 1]) (Pearson r = 0.484, *p* < 0.001). The association was further examined in interaction models to assess whether obesity indices (WC and BMI) modified the relationship between Reti-CVD and CACS.

### 3.3. Comparative Analysis of the Moderating Effects of WC and BMI

Table 2 summarizes interaction analyses across sequentially adjusted models (Models 1–4). In WC-based interaction models, the interaction term was not statistically significant in Model 1 (*p* = 0.0759), but was statistically significant in Models 2–4 (*p* = 0.0312, *p* = 0.0245, and *p* = 0.0288, respectively), with adjusted R^2^ values of 0.224, 0.258, 0.301, and 0.372, respectively. In BMI-based interaction models, the interaction term was not statistically significant in Models 1–4 (*p* = 0.3780, *p* = 0.4521, *p* = 0.5102, and *p* = 0.5381, respectively), with adjusted R^2^ values of 0.231, 0.262, 0.298, and 0.366, respectively. To quantify the magnitude of the moderation effect, standardized regression coefficients (per 1-SD increase in each variable) were derived for the fully adjusted model: the Reti-CVD ×WC interaction was β = 0.28 (95% CI, 0.03 to 0.53), indicating that the positive association between Reti-CVD and log(CACS + 1) became stronger at higher waist circumference, whereas the Reti-CVD×BMI interaction was small and non-significant (β = 0.11; 95% CI, −0.13 to 0.34). The Reti-CVD×WC interaction coefficients were positive and directionally consistent across the sequentially adjusted models (Table 2). Multicollinearity was not a major concern, as all variance inflation factors were below 5 (maximum VIF, 3.4). In the fully adjusted model, 118 participants with coronary calcification were modelled with 16 covariates (approximately 7 events per variable); accordingly, the more parsimonious Models 2 and 3 were regarded as the primary basis for inference, with Model 4 used to assess the consistency of the findings. In sensitivity analyses using logistic regression for the presence of coronary calcification (CACS > 0) as an alternative to the log-transformed linear model, the Reti-CVD×WC interaction remained significant (*p* = 0.033 unadjusted; *p* = 0.049 after adjustment for age and sex), consistent with the primary findings.

### 3.4. Visualization of Differential Predictive Patterns and ROC Analysis

**Figure 1:**
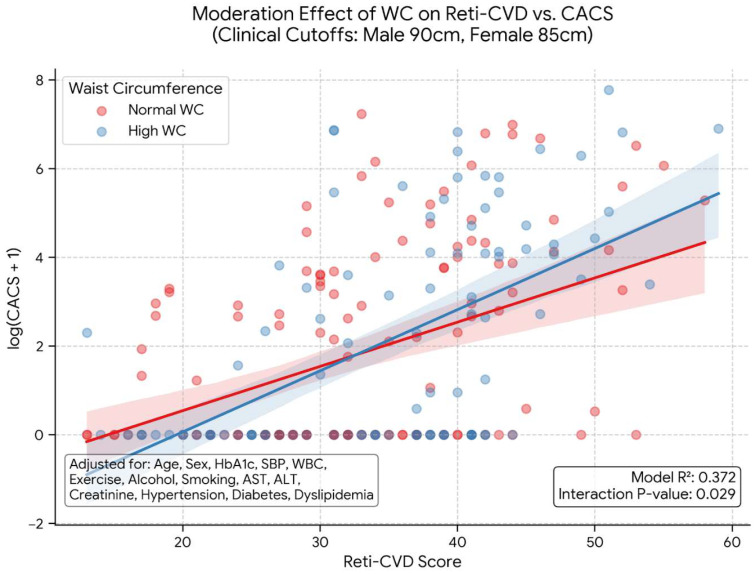
Moderation Effect of Abdominal Obesity on the Relationship Between Reti-CVD and Coronary Artery Calcium Score.

**Figure 2:**
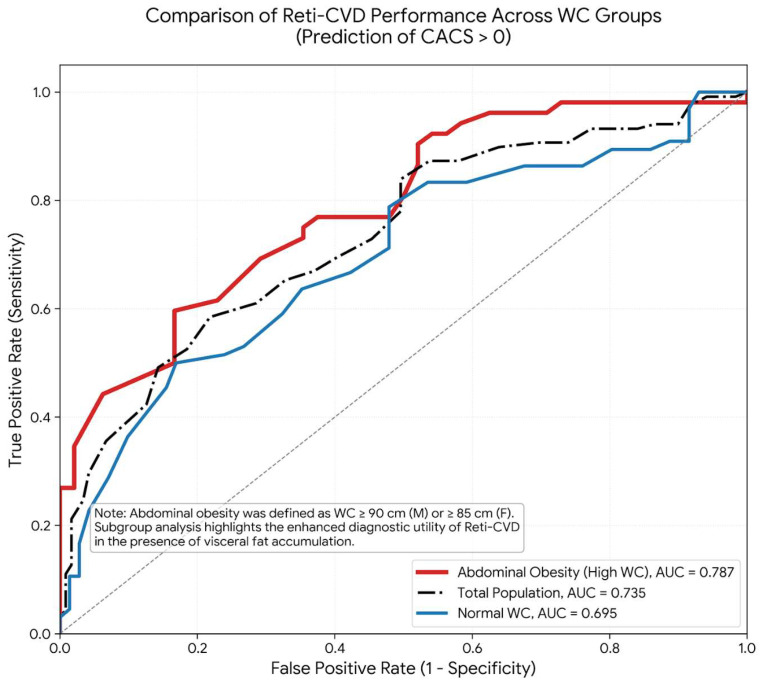
ROC Curves for Predicting Coronary Calcification (CACS > 0) Using Reti-CVD. Receiver operating characteristic (ROC) curves for predicting coronary calcification (coronary artery calcium score [CACS] > 0) using Reti-CVD. The area under the ROC curve (AUC) was 0.735 in the total population, 0.787 among participants with abdominal obesity, and 0.695 among participants with normal waist circumference (WC). The difference in AUC between WC-defined subgroups was not statistically significant (*p* = 0.182). Abdominal obesity was defined as WC ≥ 90 cm in men and ≥85 cm in women. (ROC = receiver operating characteristic; AUC = area under the ROC curve; CACS = coronary artery calcium score; WC = waist circumference).

Interaction plots were used to visualize the relationship between Reti-CVD and CACS according to obesity indices. In the WC-based analysis, the slope of the association between Reti-CVD and CACS differed between participants with abdominal obesity and those with normal WC (Figure 1). In the BMI-based analysis, the slopes were similar between BMI-defined obesity groups. The diagnostic performance of Reti-CVD for coronary calcification (CACS > 0) was evaluated using receiver operating characteristic (ROC) analysis (Figure 2). In the total population, the area under the ROC curve (AUC) was 0.735. In subgroup analyses by WC phenotype, the AUC was 0.787 in participants with abdominal obesity and 0.695 in participants with normal WC; the between-group comparison did not reach statistical significance (*p* = 0.182). Because the between-group difference in AUC was not statistically significant, this numerically higher discrimination among centrally obese participants should be interpreted with caution. When Reti-CVD was added to a baseline model comprising conventional demographic and cardiovascular risk factors, the C-statistic for the presence of coronary calcification did not change appreciably (baseline model, 0.80; baseline plus Reti-CVD, 0.80; ΔC-statistic ≈ 0.00, 95% CI −0.01 to 0.01), indicating that Reti-CVD did not provide additional discrimination beyond conventional risk factors in this cross-sectional setting. Of note, Reti-CVD obtained from a single non-invasive retinal image, without any blood sampling, achieved an AUC of 0.735, approaching that of the full multivariable risk-factor model.

## 4. Discussion

This cross-sectional study examined whether obesity indices modify the association between Reti-CVD, a deep learning-derived retinal biomarker, and coronary artery calcium score (CACS) in asymptomatic Korean adults undergoing routine health screening. Reti-CVD was positively correlated with log-transformed CACS (log[CACS + 1]) and was higher among participants with CACS > 0 than among those with CACS = 0. In interaction analyses with sequential adjustment, waist circumference (WC) modified the Reti-CVD–CACS association, whereas body mass index (BMI) did not. Notably, the WC interaction term was not statistically significant in the unadjusted model but became significant after adjustment for age and sex and remained significant in subsequent models, while BMI-based interaction terms were not significant across models. Together, these findings indicate that central adiposity captured by WC provides additional information when interpreting the relationship between Reti-CVD and coronary calcification.

Several points place these results in the context of prior work. Retinal imaging has been proposed as a practical, non-invasive approach to cardiovascular risk assessment, and Reti-CVD has demonstrated performance comparable with or superior to established risk scores in large datasets [9,10]. Our study extends this literature by focusing on subclinical coronary atherosclerosis quantified by CACS and by testing effect modification by obesity phenotype. From a clinical standpoint, WC and BMI represent distinct constructs: BMI does not distinguish between fat and lean mass or reflect fat distribution, whereas WC is more closely linked to visceral adiposity [14,15,16,17]. Central obesity has been associated with higher total and cardiovascular mortality even among adults with normal BMI [18]. Because visceral adiposity is strongly associated with cardiometabolic risk, incorporating WC phenotype may improve the interpretability of retinal biomarker outputs in health screening settings.

A biologically plausible explanation is that central adiposity is associated with metabolic and inflammatory processes that may contribute to both retinal microvascular changes and coronary atherosclerosis. Visceral fat is metabolically active and is associated with adverse adipokine and cytokine profiles and systemic inflammation [5,19]. These pathways are also linked to endothelial dysfunction and atherosclerotic processes, which may be reflected in microvascular beds such as the retinal circulation [15,20,21]. Consistent with this framework, our results suggest that the association between a retinal biomarker and coronary calcification may differ according to central adiposity phenotype. Importantly, given the observational design, these mechanisms should be considered as potential explanations rather than causal inferences.

The findings may have implications for risk stratification in primary care and screening settings where repeated CACS assessment can be constrained by radiation exposure and cost [2,3]. In practice, a retinal biomarker can be obtained from fundus photographs with minimal burden, and WC is readily measured. Integrating these measures may help identify subgroups in whom a retinal biomarker is more informative for coronary calcification risk. In ROC analyses, discrimination for CACS > 0 was numerically higher among participants with abdominal obesity than among those with normal WC; however, the between-group comparison did not reach statistical significance (*p* = 0.182). Therefore, subgroup differences in discrimination should be interpreted cautiously and may require larger samples for confirmation. The present results support a phenotype-informed approach rather than a change in clinical thresholds or a stand-alone triage strategy. The incremental clinical value of Reti-CVD should also be interpreted within these limits. In our data, adding Reti-CVD to a model of conventional risk factors did not significantly improve discrimination for coronary calcification (ΔC-statistic ≈ 0.00), which is not unexpected for a cross-sectional surrogate endpoint in a screening population in which age, sex, and metabolic factors already explain much of the calcification burden. Rather than replacing established risk factors, a retinal biomarker is best positioned as a complementary, non-invasive tool: Reti-CVD alone recovered most of the discrimination of a full risk-factor panel from a single image without blood tests, and its prospective value for predicting cardiovascular events has been demonstrated in larger cohorts [9,10]. This view is consistent with the broader movement toward multimodal and personalized cardiovascular risk assessment, in which emerging imaging-based, biological, or AI-derived markers complement, rather than supplant, traditional risk factors and simple anthropometric measures such as waist circumference [22]. Within this framework, the principal contribution of the present study is to show that the relationship between a retinal biomarker and subclinical coronary atherosclerosis is modified by central adiposity, supporting a phenotype-informed interpretation.

This study has limitations. First, the cross-sectional design precludes temporal or causal interpretation, and the single-center health screening population may limit generalizability and introduce selection bias. Second, although we applied sequential adjustment including metabolic variables, lifestyle factors, and treatment history, residual confounding remains possible. Third, Reti-CVD is an integrated AI-derived score rather than a direct quantification of individual retinal vascular parameters; this enhances practicality but limits mechanistic specificity. Fourth, subgroup analyses (including ROC comparisons) may have been underpowered to detect modest differences. Finally, the study did not evaluate longitudinal cardiovascular outcomes; future prospective studies are needed to determine whether phenotype-informed interpretation of Reti-CVD improves the prediction of clinical events beyond CACS. In addition, detailed pharmacological data were limited: although treatment for hypertension, diabetes, and dyslipidemia was captured as categorical medication-use variables and adjusted for in the fully adjusted model, the specific drug class, agent, and dose (for example, statin type and intensity) and the use of antiplatelet or other cardioprotective agents were not consistently recorded in the routine screening records. Because such therapies can influence both vascular status and coronary calcification, residual confounding by unmeasured drug effects cannot be excluded, and future studies with complete prescription data are warranted. Furthermore, Reti-CVD is a proprietary, commercially deployed algorithm whose internal architecture and weights are not publicly available; consequently, the exact score-generation process cannot be independently reproduced from this report, and we relied on the manufacturer’s validated pipeline and previously published development and validation studies [9,10,12]. Several further limitations relating to sample size, modelling, generalizability, and biomarker transparency warrant emphasis. To our knowledge, this is among the first studies to examine the relationship between a deep learning-derived retinal biomarker and coronary artery calcium in a Korean health-screening population, and the relatively modest sample size (*n* = 237) was an inherent constraint of this early, single-center experience. With 118 participants having coronary calcification and a large number of covariates in the fully adjusted model (approximately 7 events per variable), Model 4 estimates may be susceptible to overfitting; we therefore based our primary inference on the more parsimonious models, confirmed that variance inflation factors indicated no serious multicollinearity (maximum VIF, 3.4), and regard the findings as hypothesis-generating rather than definitive. In addition, although CACS is characteristically zero-inflated, and our results were robust to an alternative logistic specification, larger studies employing dedicated approaches for zero-inflated outcomes (for example, hurdle or zero-inflated models) would allow more refined modelling. Importantly, the discrimination of Reti-CVD did not exceed that of a conventional risk-factor model for this cross-sectional endpoint, so its added value should be confirmed against clinical event outcomes in prospective studies. Generalizability is also limited: the study population comprised asymptomatic Korean adults from a single screening center, and the findings may not extend to other ethnicities, healthcare settings, or higher-risk populations. Finally, beyond the proprietary nature of the algorithm noted above, detailed information on its training cohorts, calibration, and external validation is limited in the public domain; although external validation has been reported in independent cohorts including the UK Biobank and a multi-ethnic Asian population [9,10], algorithm calibration was not assessed in the present cohort, and greater transparency regarding model development and calibration would strengthen confidence in biomarker-based findings.

In conclusion, Reti-CVD is associated with coronary calcification in asymptomatic adults, and central adiposity measured by WC modifies this relationship, whereas BMI does not. Future multicenter prospective studies with longitudinal outcomes are warranted to validate these findings, clarify their generalizability, and determine the clinical value of integrating obesity phenotype into retinal biomarker-based cardiovascular risk assessment.

## 5. Conclusions

This study shows that Reti-CVD, a deep learning-derived retinal biomarker, is associated with coronary artery calcium score (CACS) in asymptomatic adults undergoing routine health screening. Waist circumference (WC), but not body mass index (BMI), modified the Reti-CVD–CACS relationship after adjustment for metabolic and lifestyle factors, indicating that central adiposity provides additional information when interpreting retinal biomarker outputs. These findings suggest that integrating WC-defined abdominal obesity into retinal imaging-based assessment may improve phenotype-informed cardiovascular risk stratification. In primary care and screening settings, combining Reti-CVD with simple anthropometric measures such as WC may support non-invasive identification of individuals who could benefit from more intensive preventive evaluation and tailored risk management strategies.

Reti-CVD is a deep learning-derived retinal biomarker computed from non-mydriatic fundus photographs. In 237 asymptomatic Korean adults undergoing routine health screening, Reti-CVD was associated with coronary artery calcium score (CACS). Waist circumference (WC)—a marker of central adiposity—significantly modified the Reti-CVD–CACS relationship after sequential adjustment for metabolic and lifestyle factors, whereas body mass index (BMI) did not. These findings suggest that incorporating WC-defined abdominal obesity may provide additional information when interpreting retinal biomarker outputs for phenotype-informed cardiovascular risk stratification.

## Figures and Tables

**Table 1:** Baseline characteristics of participants according to the presence of coronary artery calcification (*n* = 237).

Variables	CACS = 0 (*n* = 119)	CACS > 0 (*n* = 118)	*p*-Value
Age (years)	47.3 ± 6.2	54.4 ± 5.9	<0.001
Gender (Male), *n* (%)	41 (34.5)	74 (62.7)	<0.001
Body mass index (kg/m^2^)	24.4 ± 3.1	25.1 ± 3.3	0.092
Waist circumference (cm)	82.4 ± 9.2	86.6 ± 9.4	0.001
Systolic Blood Pressure (mmHg)	120.3 ± 14.1	128.7 ± 15.3	<0.001
Hypertension medication, *n* (%)	16 (13.4)	44 (37.3)	<0.001
Diabetes medication, *n* (%)	4 (3.4)	21 (17.8)	<0.001
Dyslipidemia medication, *n* (%)	15 (12.6)	27 (22.9)	0.037
Current Smoker, *n* (%)	18 (15.1)	30 (25.4)	0.048
Regular Exercise, *n* (%)	56 (47.1)	52 (44.1)	0.643
White Blood Cell (×10^3^/μL)	5.4 ± 1.3	5.8 ± 1.5	0.042
HbA1c (%)	5.6 ± 0.5	6.0 ± 0.8	<0.001
AST (U/L)	21.8 ± 7.4	24.2 ± 10.1	0.038
ALT (U/L)	23.5 ± 15.8	28.4 ± 20.2	0.041
Creatinine (mg/dL)	0.85 ± 0.16	0.91 ± 0.20	0.012
LDL-Cholesterol (mg/dL)	111.8 ± 30.2	113.1 ± 32.8	0.748
Reti-CVD score	0.09 ± 0.05	0.15 ± 0.09	<0.001

Values are means ± standard deviation or numbers (percentages). Data are presented as mean ± standard deviation or number (percentage). *p*-values are calculated using independent *t*-test for continuous variables and Chi-square test for categorical variables.

**Table 2:** Comparison of moderating effects and model explanatory power between waist circumference and body mass index.

Adjustment Models	Interaction (Reti-CVD×WC)		Interaction (Reti-CVD×BMI)	
	Interaction *p*	Adjusted R^2^	Interaction *p*	Adjusted R^2^
Model 1	0.0759	0.224	0.3780	0.231
Model 2	0.0312	0.258	0.4521	0.262
Model 3	0.0245	0.301	0.5102	0.298
Model 4	0.0288	0.372	0.5381	0.366

Values are presented as β (95% confidence interval) unless otherwise indicated. The dependent variable was log-transformed as log(CACS + 1), and adjusted R^2^ is reported for each model. Model 1 was unadjusted. Model 2 was adjusted for age and sex. Model 3 was additionally adjusted for hemoglobin A1c, systolic blood pressure, and white blood cell count. Model 4 was additionally adjusted for aspartate aminotransferase, alanine aminotransferase, serum creatinine, lifestyle factors (current smoking, current alcohol intake, and regular exercise), and history of hypertension, diabetes mellitus, and dyslipidemia treatment. Interaction models included the product term between Reti-CVD and waist circumference (Reti-CVD×WC) or body mass index (Reti-CVD×BMI) within each model. CACS = coronary artery calcium score; WC = waist circumference; BMI = body mass index.

## Data Availability

Data are available upon reasonable request.
